# A new algorithm for multiple medical image encryption based on stacked representation and block division

**DOI:** 10.1038/s41598-025-16146-7

**Published:** 2025-10-09

**Authors:** Yousef S. Alsahafi, Akram Y. Sarhan, Yasmin M. Elnabawy, Khalid M. Hosny

**Affiliations:** 1 Department of IT, College of Computing and Information Technology, University of Jeddah, Khulis, Saudi Arabia; 2 Department of CS, Faculty of Computers and Information, Suez University, Suez, Egypt; 3 Department of IT, Faculty of Computers and Information, Zagazig University, Zagazig, Egypt

**Keywords:** Multiple medical image encryption, Stacked images, Baker chaotic map, Block division, Confusion and diffusion, Engineering, Mathematics and computing

## Abstract

In the era of big data, medical imaging systems create millions of medical images daily. Securing these images during their transmission and storage is a challenging task. Encryption is a practical approach for securing medical images, as it ensures the security of these images while preserving their sensitive contents. Multiple-image encryption is preferable to single-image encryption, in which patches of medical images are encrypted simultaneously. A novel multi-medical image encryption algorithm based on stack representation and block division is proposed. During preprocessing, the medical images are stacked into a 3D cube to prepare for the encryption process. The cube experiences a block division phase, followed by individual confusion and diffusion phases for each block. These phases utilize different keys generated from a Baker chaotic map, incorporating swapping and XOR operations, which result in a fully encrypted cube. The scalability and effectiveness of the proposed algorithm are tested through experiments, revealing its promise as a secure approach for encrypting color and grayscale medical images. The proposed MIE algorithm is robust against various attacks, exhibiting superior performance in terms of speed and security compared to similar methods.

## Introduction

Advancements in data transmission and multimedia processing techniques add significant risks to the security and privacy of medical data. As a vital data type, images are essential in various fields, including electronic healthcare^1^. These images typically contain sensitive information^2^, and unauthorized access to this data could result in significant problems. Developing an efficient and safe image encryption algorithm has become a considerable concern in image processing^3^. Researchers have suggested several encryption techniques, including chaos-based^4–6^, DNA-based^7–9^, compressive sensing^10,11^, transform domain^12^, and AI-based^13,14^ approaches, among others. Hosny et al.^15^ presented an elegant and extensive survey for securing multimedia content using various encryption approaches.

Many researchers are focusing on studying MIE algorithms to secure the large number of medical images transmitted^16^. MIE enhances security by combining multiple images into a single encrypted format, increasing complexity and making unauthorized decryption more challenging. This approach provides cohesive protection that mitigates the effects of breaches, surpassing the individual safeguarding of single images in complexity. Additionally, it reduces computational expenses compared to processing each image independently.

In recent years, several notable MIE algorithms have been proposed, including^17,18^. In the first paper, Chen et al. used DNA, and the gyrator transformed with the XOR operation to design an algorithm for MIE. In the second paper, Zhang et al. used DCT, Lorenz, and logistic maps to encrypt multiple images. Zhang & Gao^19^. proposed a MIE technique based on using a scrambling-diffusion structure, bit planes, Zigzag transformation, and exclusive OR operation. Wu et al.^20^ proposed an MIE algorithm based on DNA encoding and the SHA-256 hashing algorithm. Song et al.^21^ proposed a parallel image encryption scheme using intra-bitplane scrambling and multithreaded diffusion to accelerate the encryption process. While the method targets single images, its parallel architecture and efficiency improvements are highly relevant to MIE scenarios, where speed and scalability are critical.

Perez et al.^22^ proposed an MIE algorithm based on the JTC architecture, GT, and the JGPD. Zhang & Liu^23^ developed an MIE algorithm for encrypting multiple grayscale images using SZT, the Henon chaotic map, and the 2D ZT. Kumar & Dua^24^ utilized the ESC map and DNA to develop an MIE algorithm for RGB color images. Liu et al.^25^ proposed a semantically enhanced selective image encryption scheme based on salient object detection and parallel chaos-based encryption. Although it focuses on single images, its strategy of encrypting only sensitive regions and improving efficiency via parallel processing is relevant to MIE scenarios, where both speed and intelligent resource usage are essential.

Song et al.^26^ introduced a batch image encryption scheme that utilizes cross-image permutation and bidirectional diffusion, enabling the encryption of multiple images of varying sizes without requiring preprocessing. Du et al.^27^ proposed an MIE technique, which the study implemented into face images. The study began with a face detection process to capture the desired facial information from multiple plain images that were input.

Stack Image Representation (SIR) employs a stacking approach, where images are arranged in layers along the vertical dimension, resulting in a 3D array^28^. This representation is beneficial for enhancing the security of image encryption and enabling simultaneous processing of multiple images, preserving their unique characteristics while improving efficiency and maintaining data integrity, making it a valuable strategy for robust multidimensional image handling.

Despite their efficiency, existing MIE schemes have certain limitations. Certain methods are limited to encrypting a specific number of images simultaneously rather than accommodating an arbitrary number. Most algorithms are primarily tested on standard grayscale images, with very few explicitly tailored for medical image encryption. Others work with either grayscale or color images only. This paper, therefore, introduces an encryption scheme designed explicitly for multiple medical images. This paper’s primary contributions are delineated as follows:

1. The proposed MIE algorithm encrypts a massive number of medical images simultaneously and is designed to effectively encrypt grayscale and color images, showcasing its adaptability and broad applicability.
2. It creates interconnected cipher images within the same stack, where if one cipher image is lost, the remaining cipher images in the stack can help recover partial information from the lost image.
3. MIE is implemented using multilayer encryption to enhance security, including subdivision, 3D transformation, block segmentation, confusion, and diffusion processes. Subdivide the images and apply 3D transformations to increase the complexity of the decryption process.
4. Applying block segmentation-based encryption to disrupt the intra-correlation between adjacent pixels and utilize different keys generated from a Baker chaotic map, incorporating swapping and XOR operations to apply confusion and diffusion processes for each block.

This multi-stage approach provides high security and complexity, making unauthorized decryption attempts for multiple medical images challenging. It integrates stacking randomization, confusion, and diffusion, supported by chaotic maps and stacking representation.

 The paper is organized as follows: the proposed MIE algorithm is presented in Sect. 2. The validity and the effectiveness of the proposed method are presented in Sect. 3. Experiments, results, and the discussion are included in Sect. 4. Finally, the conclusion is given in Sect. 5.

## Proposed method

### Encryption scheme for color images

The encryption process for multiple images involves several sequential steps, each contributing to the security of the images. The entire process can be broken down into the following stages, as illustrated in the diagram below.

#### Crop process

For each original image $\:I\:$with dimension $\:W\times\:H\times\:D\:$and desired crop size $\:{w}_{c}\:and\:{h}_{c}$:

1. Split each image into RGB channels $\:{I}_{r},{I}_{g},\:{I}_{b}$, each of which has a size of $\:W\times\:H$.

2. Determine the number of crops in both the width and height directions.
  1 $$\:{N}_{x}=\left[\:\frac{W}{{w}_{c}}\:\right],\:{N}_{y}=\left[\:\frac{H}{{\:h}_{c}}\:\right]\:$$

Where $\:{N}_{x}$ represents the number of crops along the horizontal direction, and $\:{N}_{y}\:$represents the number of crops along the vertical direction.

3 For each crop $\:(i,j)$ operation:

- Determine the coordinates of each crop.

$$\:{x}_{s}=i\cdot \:{w}_{c}\:,\:{x}_{e}={x}_{s}+\:{w}_{c}$$

$$\:{y}_{s}=j\cdot \: {h}_{c}\:,\:{y}_{e}={y}_{s}+\:{h}_{c}$$

Where,

2 $$\:i\in\:\left[0,\:{N}_{x-1}\right]\:,\:j\in\:[0,{N}_{y-1}]$$

Where $\:i$ denotes the horizontal index and $\:j\:$denotes the vertical index. $\:{x}_{s},\:\:{x}_{e}\:$are the start and end coordinates along the horizontal direction, and $\:{y}_{s},\:\:{y}_{e}\:$along the vertical direction.

4. Use the calculated coordinates to extract crops for each channel:

$$\:{I}_{rc}\left(i,j\right)={I}_{r}\left[{\:y}_{s}:{y}_{e}\:,\:{x}_{s}:{x}_{e}\:\right]$$

$$\:{I}_{gc}\left(i,j\right)={I}_{g}\left[{\:y}_{s}:{y}_{e}\:,\:{x}_{s}:{x}_{e}\:\right]$$

3 $$  \:{I}_{bc}\left(i,j\right)={I}_{b}[{\:y}_{s}:{y}_{e}\:,\:{x}_{s}:{x}_{e}\:]$$

#### Stacking process

After cropping all input images into smaller crops, the stacking process proceeds as follows:

1. Determine the total number of 2D crops.

4 $$\:N={[{N}_{x}+{N}_{y}]}^{2}\times\:n$$

Where $\:n$ is the total number of input images.

2. Initialize $\:cube$ of size $\:N\times\:{w}_{c}\times\:{h}_{c}$, and stack the crops $\:{C}_{l}$ onto it.

$$\:cube\left[l,a,b\right]={C}_{l}$$

where:

5 $$\:l\in\:\left[1,N\right],\:a\in\:\left[1,{w}_{c}\right],\:b\in\:[1,{h}_{c}]$$

3. Repeat steps 1 and 2 for each channel.

#### Block division

1. Let the block size be $\:{b}_{x},{b}_{y},\:and\:{b}_{z}$. Determine the number of blocks that fit along all dimensions:

6 $${N}_{b}=\left(\: \lfloor\frac{{w}_{c}}{{b}_{x}}\rfloor \:\times\: \lfloor \frac{{h}_{c}}{{b}_{y}} \rfloor\times\:\: \lfloor \frac{N}{{b}_{z}}\rfloor\:\right)$$

2. Iterate $\:{N}_{b}$ over the $\:cube$ to extract blocks by slicing them. For each iteration, extract a block as follows:

$$\:block=cube\:\left[{\:d}_{x}\::{d}_{x}+{b}_{x}\:,\:{d}_{y}\::{d}_{y}+{b}_{y}\:,\:{d}_{z}\::\:{d}_{z}+{b}_{z}\right]$$

where:

$$\:{d}_{x}\in\:\left[0\:,\:{w}_{c}\:,\:{b}_{x}\right]$$

$$\:{d}_{y}\in\:\left[0\:,\:{h}_{c}\:,\:{b}_{y}\right]$$

7 $$\:{d}_{z}\in\:\left[0\:,\:N\:,\:{b}_{z}\right]$$

3. Repeat steps 1 and 2 for each channel.

#### Permutation and substitution

In this process, pixels in each block are permuted and substituted using different keys according to the following steps.

1. Generate two keys for each block using $\:\alpha\:\:and\:\beta\:$ parameters, which are calculated as follows:

$$\:{\upalpha\:}=\frac{(\sum\:block)+(W\times\:{w}_{c}\times\:{b}_{x})}{{2}^{23}+\left(H\times\:{h}_{c}\right)}$$

8 $$\:\beta\:=\frac{{e}^{\left(\alpha\:\:mod\:2\right)}}{10}$$

2. Insert $\:\alpha\:\:and\:\beta\:$ parameters into the Baker map defined by^29^ to generate the keys $\:({X}_{p},\:{Y}_{s})$.

9 $$\:\left({X}_{n+1},{Y}_{n+1}\right)=\:\left\{\begin{array}{c}\left(\frac{{X}_{n}}{\alpha\:},\alpha\:{Y}_{n}\right),\:0<X\:\le\:\alpha\:\\\:\left(\frac{{(X}_{n}-\alpha\:)}{(1-\alpha\:)},\:\left(1-\alpha\:\right){Y}_{n}+\left(1-\alpha\:\right)\right),\:\alpha\:<x\le\:1\end{array}\right.$$

3. Apply the permutation process for each block. In this phase, the pixel positions across the block are permuted to achieve a more complex data mixing. In this step $\:block$ cube is scrambled by using a sequence $\:{X}_{p}$ as follows:

i. Flattening *block* cube into a 1D vector $\:v$, as follows:

10 $$\:v\left[r\right]=block[\:{b}_{x}\:,{b}_{y}\:,{\:b}_{z}\:]$$

where $\:r$ is a linear index in the 1D vector, and let $\:{v}_{n}=\:{b}_{x}\times\:{b}_{y}\times\:{b}_{z}$.

ii. Swapping values in the vector as follows:

$$\:let,\:{K}_{1}=\lfloor{X}_{p}\rfloor\:mod\:{v}_{n}$$

$$\:then,$$

11 $$\:\:v\left[r\right]\leftrightarrow\:v\left[{K}_{1}\left[r\right]\right],\:r\in\:\left[0,{v}_{n}\right]$$

4. Apply the substitution process for each block. In this phase, the pixel values are adjusted to obscure their original content using $\:{Y}_{s}$, where the values of the vector $\:v$ is changed as follows:
  i. The bitwise XOR operation is performed as follows.

$$\:let,\:{K}_{2}=\lfloor{Y}_{s}\rfloor\:mod\:{v}_{n}$$

$$\:then,$$

12 $$\:\:v\left[r\right]=\text{v}\left[r\right]\oplus\:{K}_{2},\:\text{r}\in\:[0,{v}_{n}]$$

ii. Reshaping of vector $\:v$ is performed to obtain a final encrypted $\:block$ cube, as follows.

13 $$\:block\left[\:{b}_{x}\:,{b}_{y}\:,{\:b}_{z}\:\right]=\:v\left[r\right]$$

5. Repeat steps from 1 to 4 are applied to each block separately, obtaining a total $\:{N}_{b}$ encrypted blocks.
6. Reconstruct the stack from the encrypted blocks to obtain the encrypted stack cube.
7. Repeat steps 1 to 6 for each channel, resulting in three RGB-encrypted stacked cubes.
8. Merge the RGB encrypted stacked cubes to produce the final cipher stacked image.

**Fig. 1:**
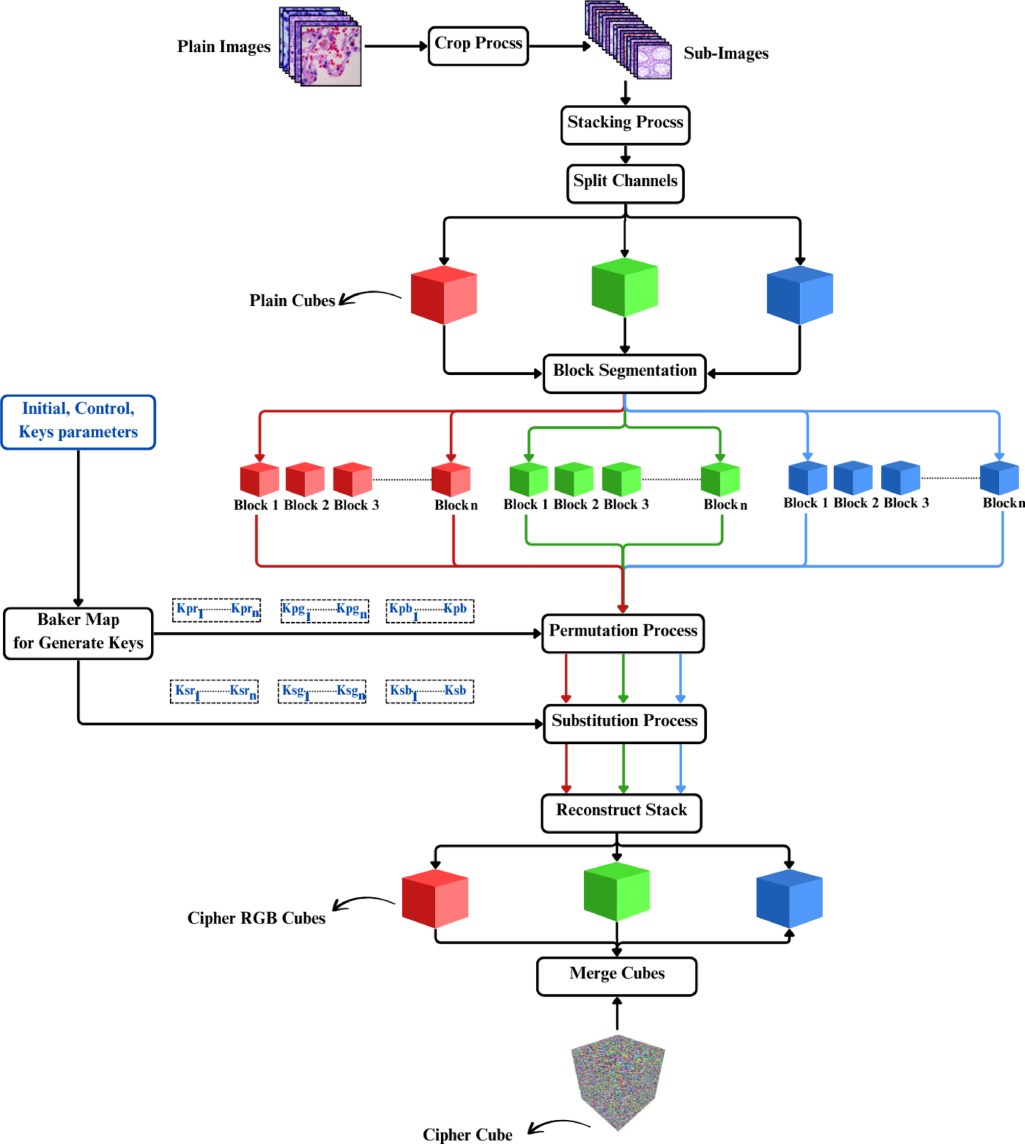
Encryption Process.

The entire encryption process is shown in Fig. 1.

### Illustrative example for one channel

We use 64 images from the Multi-Cancer Dataset—8 Types of Cancer Images dataset. This dataset contains images of various cancer types, compiled for research and analysis purposes. It encompasses 8 main cancer classes and 26 subclasses, offering a comprehensive resource for medical image classification and machine learning applications. Given that the number of input images is $\:n=64$, where the size of each image is $\:W=512,\:H=512$ and crop size $\:{w}_{c}=256,\:{h}_{c}=256$. Determine the number of crops along the width $\:{N}_{x}$ and along the height $\:{N}_{y}$:

14 $$\:{N}_{x}=\left[\:\frac{512}{256}\:\right]=2,\:{N}_{y}=\left[\:\frac{512}{256}\:\right]=2$$

The grid consists of a $\:2\times\:2$ matrix of crops, with each crop defined by its coordinates $\:{x}_{s},{y}_{s},{x}_{e},{y}_{e}$, Which results in Table 1:

**Table 1:** Example of the result of the crop process.

Crop index $\:(\varvec{i},\varvec{j})$	Coordinates	Crop Size
$\:\left(\text{0,0}\right)$	$\:(0,\:0,\:256,\:256)$	$\:256\times\:256$
$\:\left(\text{1,0}\right)$	$\:(256,\:0,\:512,\:256)$	$\:256\times\:256$
$\:\left(\text{0,1}\right)$	$\:(0,\:256,\:256,\:512)$	$\:256\times\:256$
$\:\left(\text{1,1}\right)$	$\:(256,\:256,\:512,\:512)$	$\:256\times\:256$

**Fig. 2:**
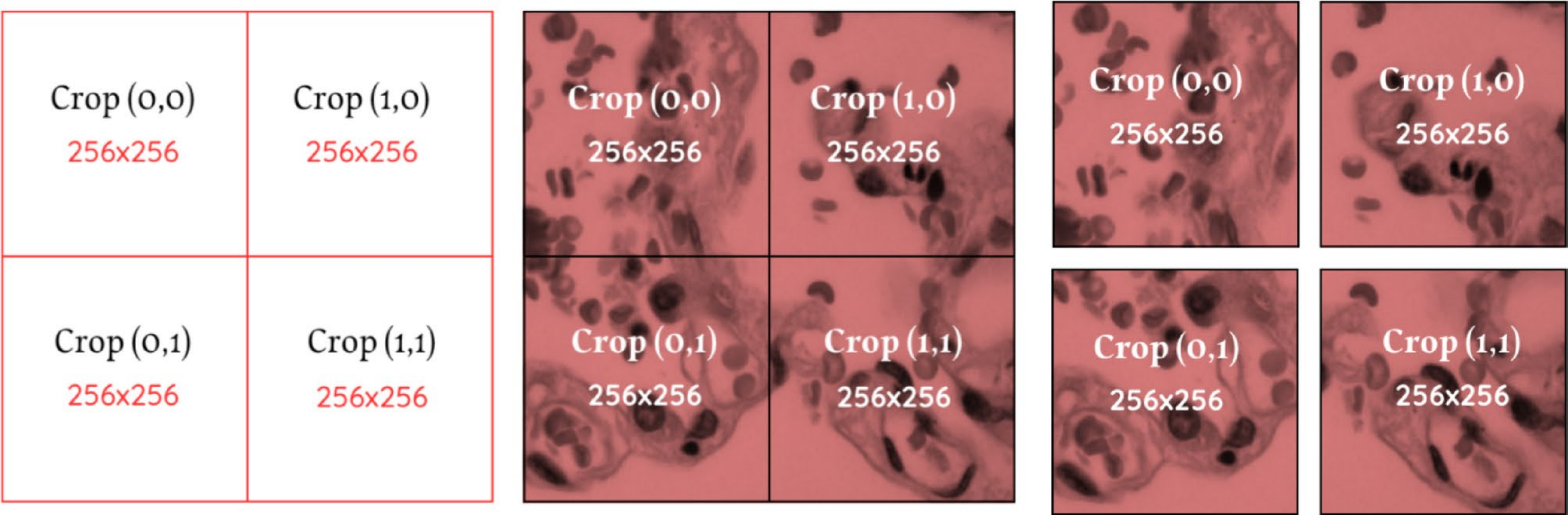
Crop process of the illustrative example for one channel. **(a)** crop frame. **(b)** original image with the crop frame. **(c)** The result of the crop process.

Figure 2 illustrates the crop process for the illustrative example of one image. The previous steps applied to all input images resulted in a total number of crops $\:N:$

15 $$\:N={\left[2+2\right]}^{2}\times\:64=256$$

**Fig. 3:**
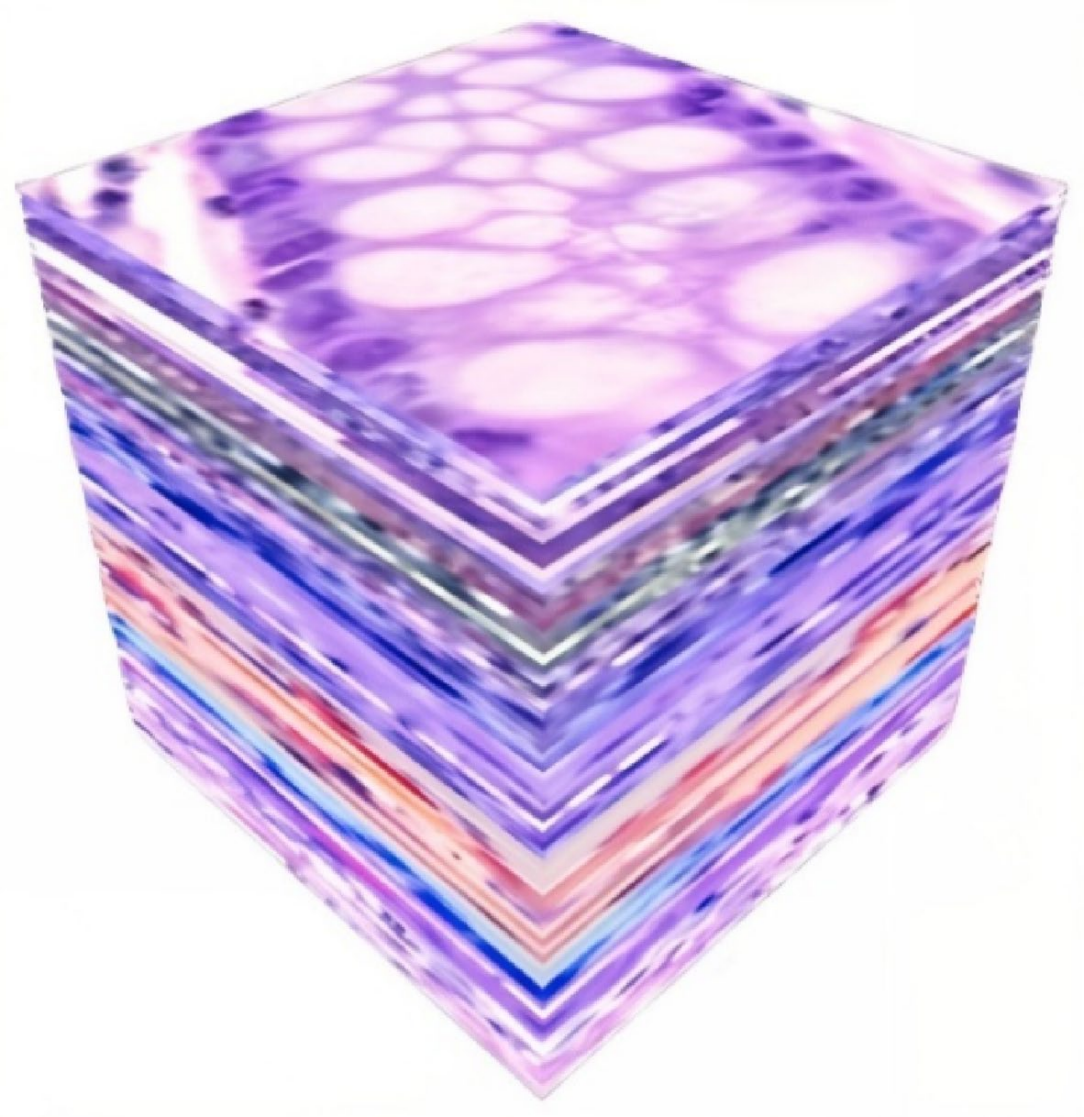
The result of the stacking process. The authors created the 3D visualization by using Python 3.9.12 and Plotly v6.2.0 (https://plotly.com/python/).

The total of 256 crops is stacked together, resulting in a 3D cube of size $\:256\times\:256\times\:256$. Figure 3 shows the result of the stack process of the illustrative example. In the block segmentation process, let the block size be $\:\left(128,\:128,\:128\right)$ then, the number of blocks that fit along each dimension is:

16 $${N}_{b}=\left(\:\lfloor \frac{256}{128}\rfloor \:\times\:\: \lfloor \frac{256}{128}\rfloor \times\:\: \lfloor\frac{256}{128}\rfloor \:\right)=8$$

**Fig. 4:**
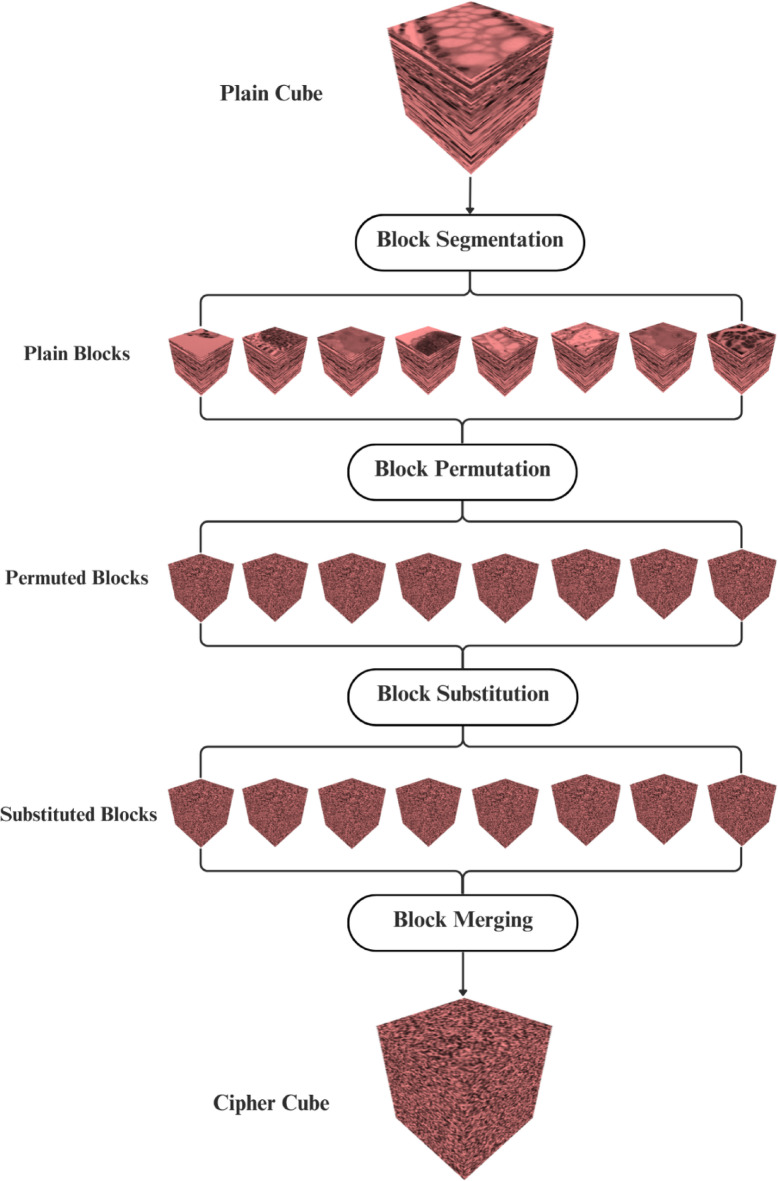
The result of the block segmentation, permutation, and substitution processes for the R-channel. The authors created the 3D visualization by using Python 3.9.12 and Plotly v6.2.0 (https://plotly.com/python/).

Figure 4   shows the result of the block segmentation process of the illustrative example, as well as the result of the permutation and substitution processes.

Table 2 is the representation for visualizing the block extraction for a total of 8 blocks:

**Table 2:** Example of the result of the block extraction process.

Blocks	x-range	y-range	z-range	Extracted Block cube
**Block 1**	$\:0:128$	$\:0:128$	$\:0:128$	$\:cube\left[0:128,\:0:128,\:0:128\right]$
**Block 2**	$\:128:256$	$\:0:128$	$\:0:128$	$\:cube\left[128:256,\:0:128,\:0:128\right]$
**Block 3**	$\:0:128$	$\:128:256$	$\:0:128$	$\:cube\left[0:128,\:128:256,\:0:128\right]$
**Block 4**	$\:128:256$	$\:128:256$	$\:0:128$	$\:cube\left[128:256,\:128:256,\:0:128\right]$
**Block 5**	$\:0:128$	$\:0:128$	$\:128:256$	$\:cube\left[0:128,\:0:128,\:128:256\right]$
**Block 6**	$\:128:256$	$\:0:128$	$\:128:256$	$\:cube\left[128:256,\:0:128,\:128:256\right]$
**Block 7**	$\:0:128$	$\:128:256$	$\:128:256$	$\:cube\left[0:128,\:128:256,\:128:256\right]$
**Block 8**	$\:128:256$	$\:128:256$	$\:128:256$	$\:cube\left[128:256,\:128:256,\:128:256\right]$

**Fig. 5:**
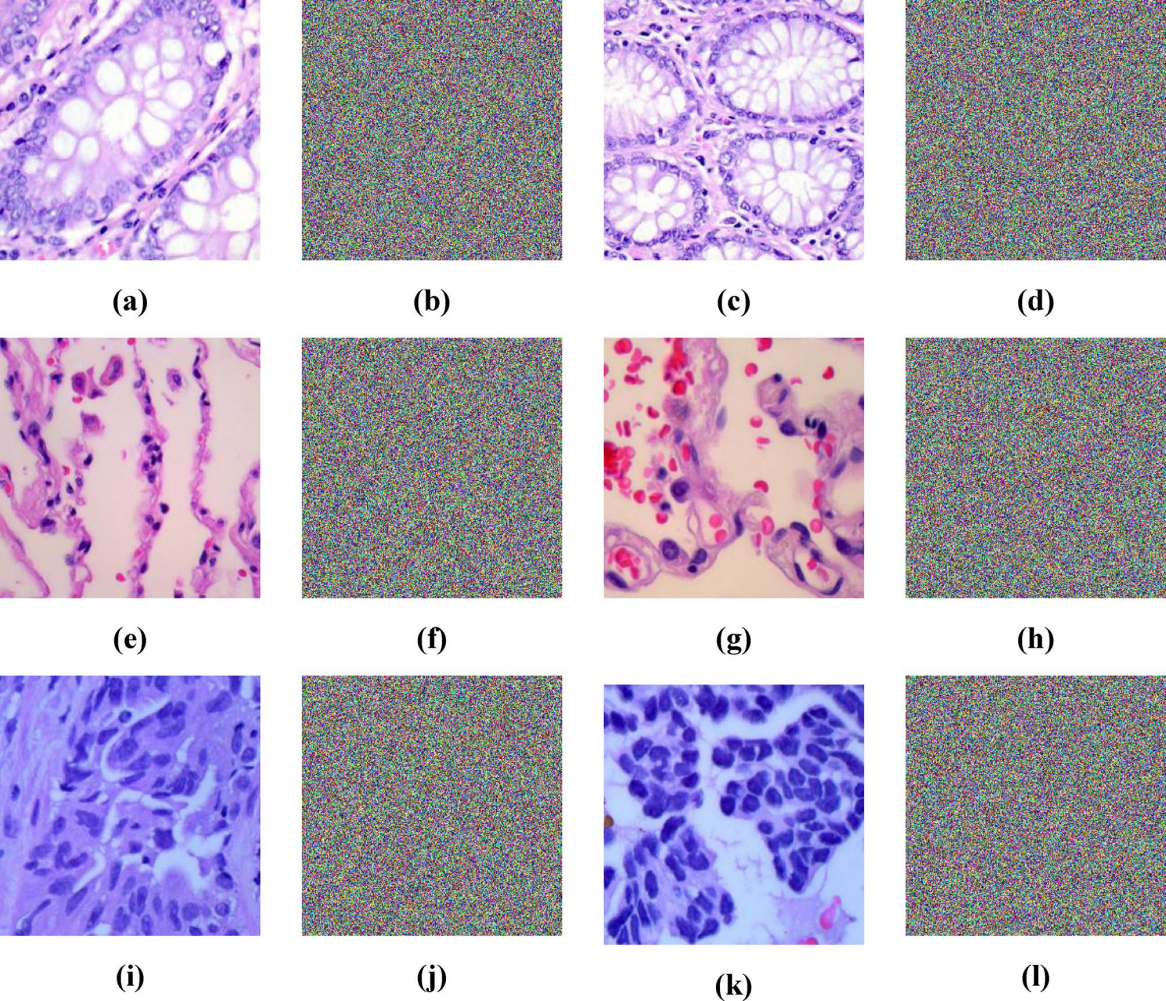
**(a**,** c**,** e**,** g**,** i**,** k)** Examples of plain images from the mentioned dataset and **(b**,** d**,** f**,** h**,** j**,** l)** their corresponding encrypted images.

Examples of plain images from the mentioned dataset and their corresponding encrypted images are shown in Fig. 5.

### Decryption process

**Fig. 6:**
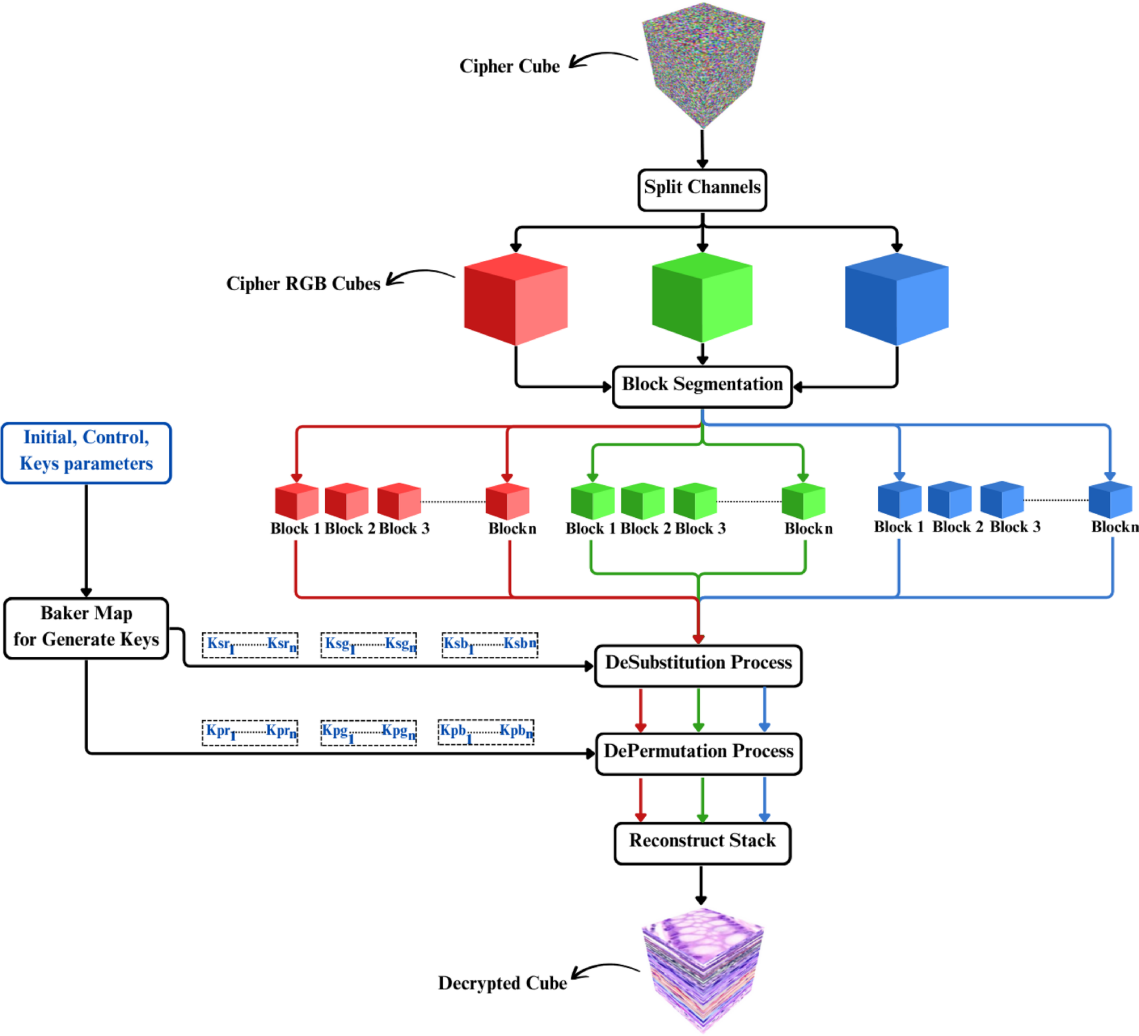
Decryption Process.

The encrypted images are divided into sub-images and stacked into a 3D cube in the decryption process. Block segmentation is then performed to prepare the images for reversing the encryption process. Figure 6 illustrates the entire decryption procedure.

## Performance analysis

Three experiments were conducted to evaluate the proposed scheme’s performance. Table 3 provides details of the images, cubes, and blocks used in these experiments.

**Table 3:** Experiments information.

	Experiments		
	Experiment 1	Experiment 2	Experiment 3
**Number of images**	64	32	16
**Image size**	$\:512\times\:512$	$\:256\times\:256$	$\:128\times\:128$
**Number of crops**	256	128	64
**Cube size**	$\:256\times\:256\times\:256$	$\:128\times\:128\times\:128$	$\:64\times\:64\times\:64$
**Block size example**	$\:128\times\:128\times\:128$	$\:32\times\:32\times\:32$	$\:8\times\:8\times\:8$
**Number of blocks**	8	64	512

### Key space

A valid image cryptosystem requires a key space large enough to render standard attacks, such as brute-force, infeasible. The key space represents the complete set of possible keys available for encryption/decryption. The secret key mainly consists of the initial values ($\:u,t)\:$and the control parameter $\:\left(\rho\:\right)\:$and the keys parameter $\:(\alpha\:,\beta\:)$. Suppose the control parameter initial values and key parameter precision are$\:{10}^{-15}$. Thus, the corresponding key space value is $\:{\left({10}^{15}\right)}^{5\times\:{N}_{b}}\:$where the minimum $\:{N}_{b}$ value is equal to 2. So, the minimum key space value for the scheme is approximately $\:{\left({10}^{15}\right)}^{5\times\:2}={10}^{150}\approx\:\:{2}^{498.29}$ which is greater than $\:{2}^{128}$. Hence, the scheme has a highly adequate key space, therefore being resilient toward brute-force attacks. Table 4 lists the key spaces of the three experiments used.

**Table 4:** Key Pace analysis.

Experiments	Key Space
**Experiment 1**	$\:{\left({10}^{15}\right)}^{5\times\:8}$
**Experiment 2**	$\:{\left({10}^{15}\right)}^{5\times\:64}$
**Experiment 3**	$\:{\left({10}^{15}\right)}^{5\times\:512}$

### Key sensitivity

**Fig. 7:**
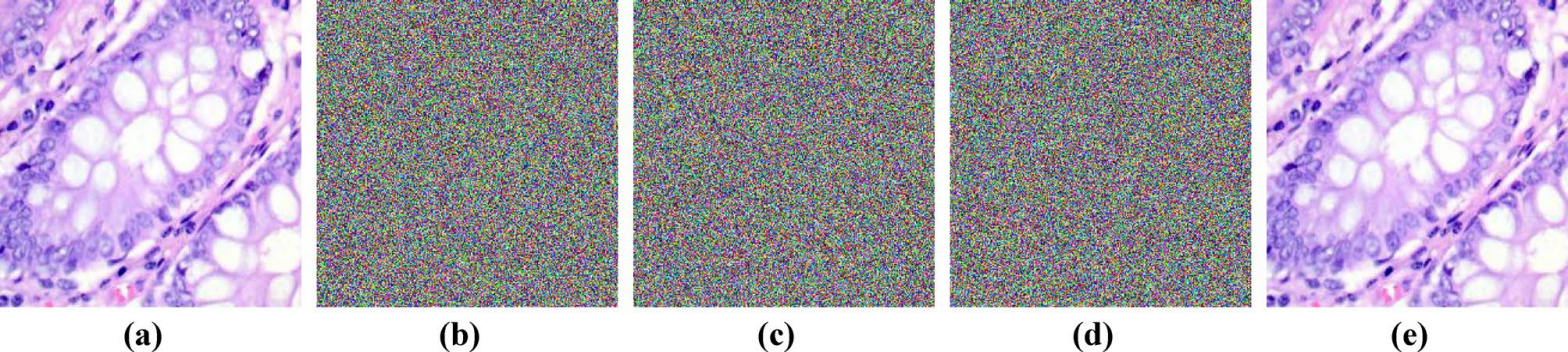
Key Sensitivity. **(a)** Plain image. **(b)** Cipher Image “$\:{c}_{1}$” using $\:{Key}_{1}$. **(c)** Cipher Image “$\:{c}_{2}$” using $\:{Key}_{2}$. **(d)** Decrypted Image c1 using the modified $\:{Key}_{2}$. **(f)** Decrypted Image $\:{c}_{1}$using the original $\:{Key}_{1}$.

The secret key should be completely different when generated using different initial conditions, especially when the difference between these initial conditions is minimal. To carry out the key sensitivity analysis, there are two primary steps. The first one, $\:{Key}_{1}$, is generated with the initial conditions $\:{x}_{0},\:{y}_{0}\:$and used to encrypt the plain image, resulting in a cipher image $\:{c}_{1}$. In the second step, we change the initial conditions to $\:{x}_{0}+{10}^{-10},\:{y}_{0}+{10}^{-10}$, then generate $\:{Key}_{2}$ and use it to encrypt the same plain image, resulting in a cipher image $\:{c}_{2}$. Finally, we decrypt the two encrypted images with the original $\:{Key}_{1}$. Figure 7 illustrates the results of these processes.

### Entropy

Entropy, as defined by^30^, quantifies an image’s level of randomness or unpredictability. It is a metric to assess how much information is hidden or concealed within the image. The entropy of the encrypted image was calculated and compared to that of the original image. The encrypted images showed a significant increase in entropy, indicating a high level of randomness and, thus, enhanced security. The amount of entropy $\:H$ of an image is determined by utilizing the following formula:

17 $$\:H=-{\sum\:}_{i}^{N-1}{p}_{i}{\text{log}}_{2}\left({p}_{i}\right)$$

Where $\:N$ is the number of possible pixel values and $\:{p}_{i}$, is the probability of occurrence of the $\:i-th$ pixel value in the image. Ideally, the information entropy for a grayscale image is 8. Table 5 provides an entropy of the three implemented experiments after encryption. An entropy value of $\:7.9999$ bits per pixel in the encrypted image is close to the theoretical ideal value. The high entropy of the encrypted image suggests that the pixel values are spread roughly uniformly. Such high entropy demonstrates that the encryption process has been highly effective in randomizing pixel values and concealing the original image content.

**Table 5:** Entropy analysis.

	Entropy			
	*R*-Channel	G-Channel	B-Channel	Mean
**Experiment 1**	7.99999	7.99999	7.99997	7.99998
**Experiment 2**	7.99991	7.99990	7.99990	7.99990
**Experiment 3**	7.99926	7.99936	7.99934	7.99932

### Histogram analysis

**Fig. 8:**
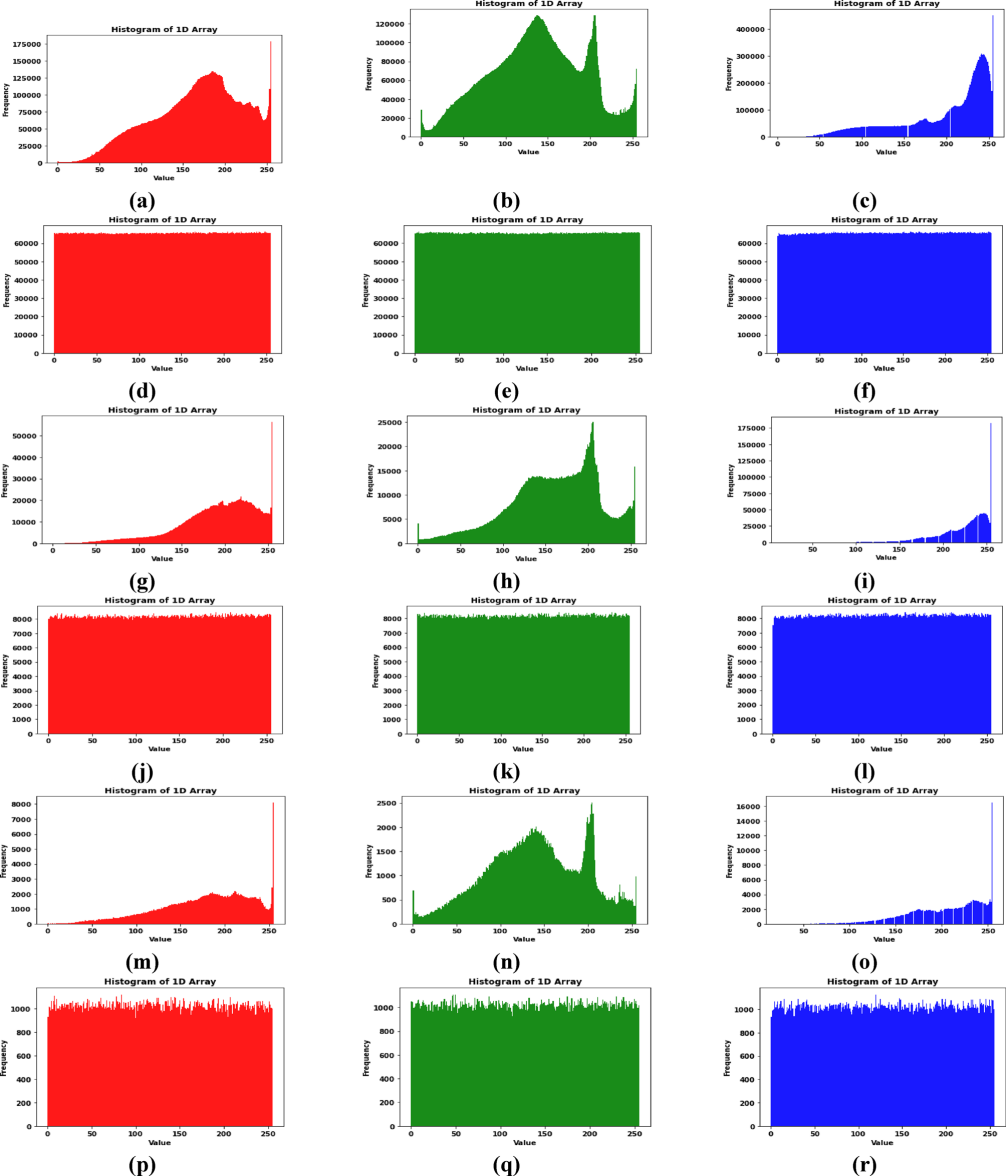
Histogram for **(a**,** b**,**c)**, **(g**,** h**,**i)**,** (m**,** n**,**o)** Experiment 1, Experiment 2, and Experiment 3 plain images respectively. **(d**,** e**,**f)**, **(j**,** k**,**l)**,** (p**,** q**,**r)** Experiment 1, Experiment 2, and Experiment 3 cipher images respectively.

The histograms of the original and encrypted images were compared to assess the effectiveness of the encryption in terms of pixel value distribution. The encrypted images exhibited uniform histograms, indicating that pixel values were effectively scrambled and that the encryption successfully masked the original image data. Figure 8 displays the histograms of the cipher cubes of the three experiments.

### Time analysis

The efficiency of the designed scheme is directly reflected in the speed of the encryption process. To evaluate this, three image sets with dimensions of $\:128\times\:128$, $\:256\times\:256$, and $\:512\times\:512$ were used. The average encryption times for these operations are presented in Table 6. The experiments were conducted on a system with an Intel Core i5-9300 H CPU (2.40 GHz, 4 cores, 8 threads) and Windows 11 OS. The average encryption time per image and channel demonstrates the method’s efficiency, with reasonable execution times across test cases. These results suggest that the proposed scheme is computationally feasible for practical applications; however, further optimization, such as parallel processing, could enhance its performance.

**Table 6:** Encryption time Analysis.

		Number of images	Size of each image	Total time for all images (s)	Average time for each image (s)	Average time for each channel (s)
**Experiment 1**		64	512$\:\times\:$512$\:\times\:$3	78	1.2	0.4
**Experiment 2**		32	256$\:\times\:$256$\:\times\:$3	10	0.3	0.1
**Experiment 3**		16	128$\:\times\:$128$\:\times\:$3	4.8	0.3	0.1

### Time complexity

The proposed MIE algorithm consists of four main stages: cropping, 3D stacking, block division, and block-wise encryption. The cropping and stacking steps are linear concerning the number of input images $\:n$, resulting in a complexity of $\:O\left(n\right)$. During encryption, each block of fixed size$\:\:b\times\:b\times\:b$ undergoes permutation and substitution, with complexity $\:O\left({b}^{3}\right)$ per block. Since the block size $\:b$ is small and constant in practice, the overall time complexity becomes $\:O(n\cdot\:{b}^{3})$, which effectively behaves as $\:O\left(n\right)$. This demonstrates the method’s scalability and computational efficiency in real-world implementations.

### Correlation analysis

An effective encryption technique should eliminate the correlation between diagonal, horizontal, and vertical pixels, ensuring that the pixel relationships in the plain images are completely obscured, thereby enhancing the security of the encrypted images and preventing statistical attacks. The correlation $\:{r}_{i,j}$ of 10,000 randomly selected adjacent pixel pairs $\:(i,j)$ from both the plain and cipher images was estimated using the following equations:

18 $$\:{r}_{i,j}=\frac{Co\left(\right(i-Co\left(i\right)\left(j-Co\left(j\right)\right))}{\sqrt{D\left(i\right)D\left(j\right)}}$$

Where:

19 $$\:Co\left(i\right)=\frac{1}{N}\sum\:_{n=1}^{N}{i}_{n},D\left(i\right)=\frac{1}{N}\sum\:_{n=1}^{N}{{(i}_{n}-Co\left(i\right))}^{2}$$

**Fig. 9:**
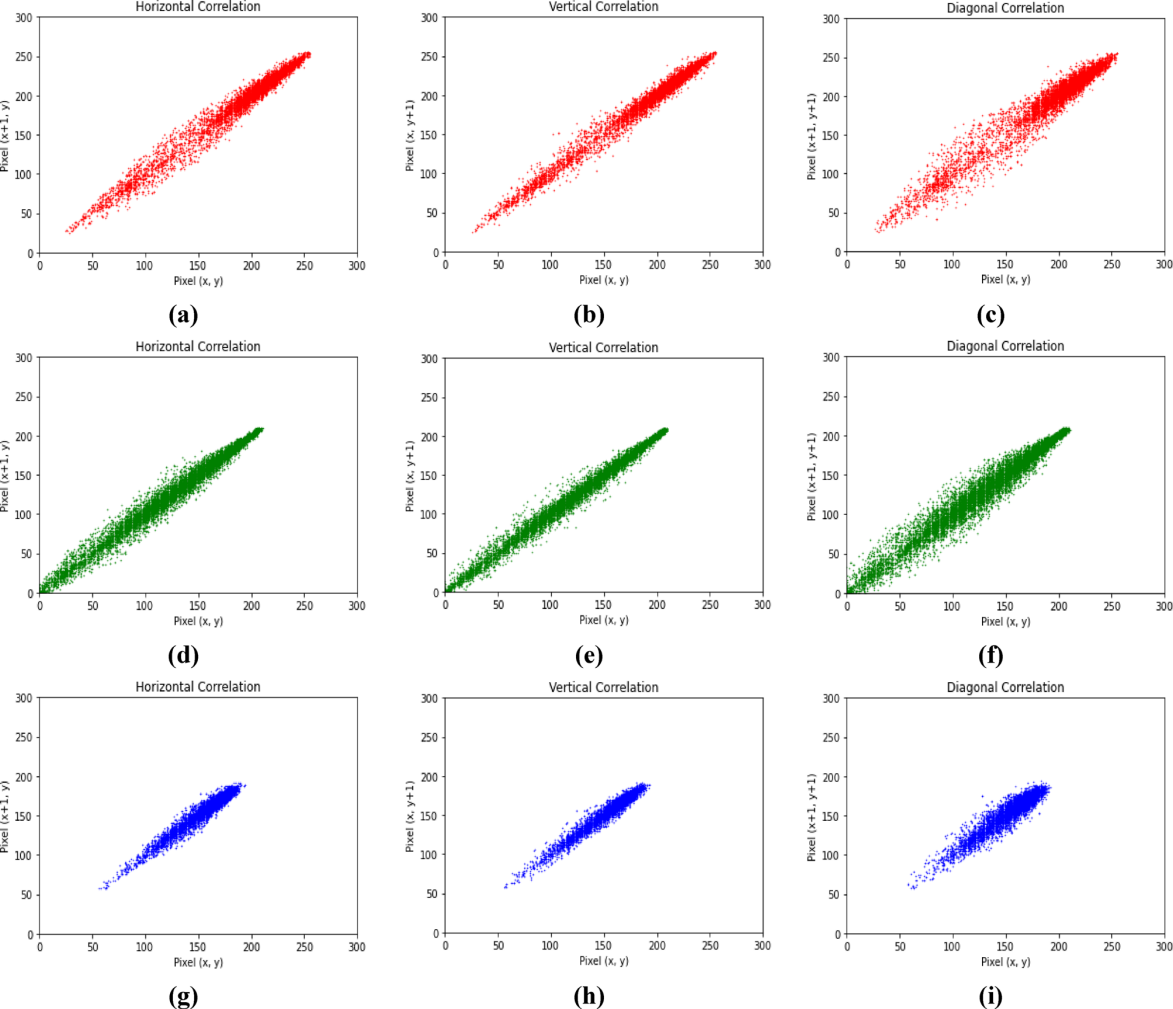
Correlation for three plain channels in the three directions.

**Fig. 10:**
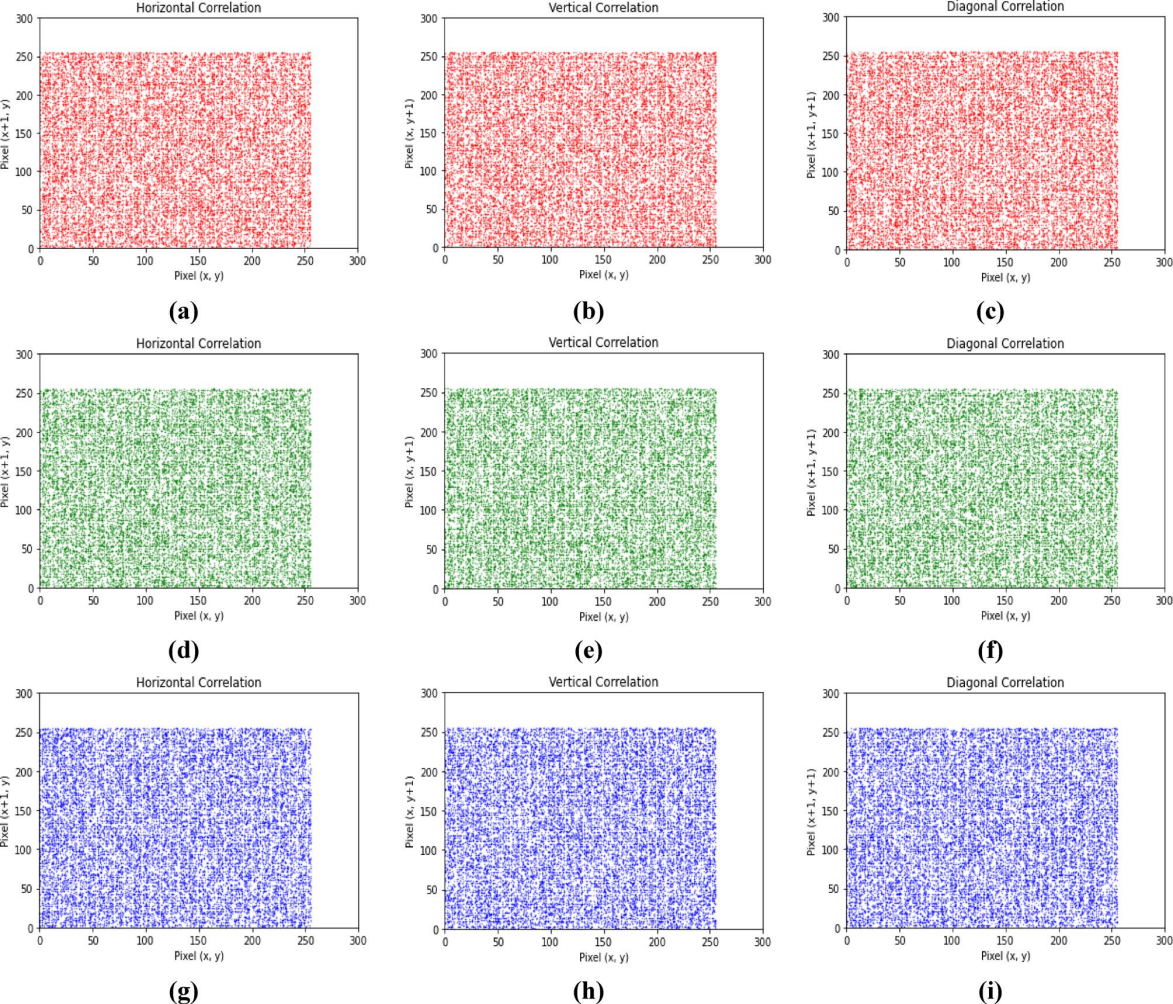
Correlation for three cipher channels in the three directions.

In Experiment 2, Figs. 9 and 10 present the distributions of pixels of the RGB channels of the color plain and corresponding cipher images along three orientations. Table 7 identifies the correlation coefficients of the RGB channels of the plain and corresponding cipher color images neighboring pixel values in three orientations. All the results of the cipher RGB channels are approaching 0, exhibiting reduced inter- and intra-channel correlation, and the pixels became highly dissimilar to each other.

**Table 7:** Correlations values of the color image.

Experiments	Direction	Plain Image			Cipher Image		
		R	G	B	R	G	B
**Experiment 1**	**H**	0.95913	0.96189	0.92644	−0.00037	−0.00072	0.00005
	**V**	0.94579	0.94903	0.90687	−0.00005	0.00054	0.00064
	**D**	0.91314	0.91811	0.85021	0.00049	−0.00002	−0.00029
**Experiment 2**	**H**	0.92867	0.94625	0.85198	−0.00130	0.00063	0.00030
	**V**	0.92158	0.94130	0.83764	0.00091	−0.00045	−0.00105
	**D**	0.86777	0.89938	0.73514	−0.00009	−0.00057	0.00134
**Experiment 3**	**H**	0.81631	0.78372	0.70706	−0.0016	0.00081	0.00224
	**V**	0.85295	0.82589	0.75617	−0.00023	−0.00097	0.00034
	**D**	0.69567	0.65114	0.54495	0.00073	−0.00099	0.00009

### Differential attack analysis

Metrics include the Unified Average Changing Intensity (UACI) and the Number of Pixels Change Rate (NPCR), which are used to quantify the resistance to different attacks. These metrics measure the differences between two encrypted images obtained by slightly altering the original image. Consequently, even minor pixel alterations in the original image substantially impact the encrypted image^31^. The following are the definitions of UACI and NPCR:

20 $$\:\left\{\begin{array}{c}UACI=\frac{1}{w\times\:h}\times\:\sum\:_{x=0}^{w}\sum\:_{y=0}^{h}\frac{\left|{C}_{1}\left(x,y\right)-{C}_{2}\left(x,y\right)\right|}{255}\times\:\:100\text{\%}\\\:NPCR=\frac{1}{w\times\:h}\times\:\sum\:_{x=0}^{w}\sum\:_{y=0}^{h}D\left(x,y\right)\times\:\:100\text{\%}\end{array}\right.$$

$$\:where$$

21 $$\:D\left(x,y\right)=\left\{\begin{array}{c}0,\:{C}_{1}\left(x,y\right)={C}_{2}\left(x,y\right)\:\\\:1,\:{C}_{1}\left(x,y\right)\ne\:{C}_{2}\left(x,y\right)\:\end{array}\right.$$

Where $\:{C}_{1},\:\text{a}\text{n}\text{d}\:{C}_{2\:}$, are two cipher images derived from a plain image with a single random pixel variation, while $\:\left(w,h\right),\:$denotes the number of rows and columns. Table 8 shows NPCR and UACI values. Suppose the resulting cubes from encrypting two virtually similar cubes show an NPCR close to 100% and a UACI larger than 33%^32^. In this case, the encryption method can be considered strong since a minor change in the algorithm’s input results in a drastically different output. The results indicate that our method exhibits resilience against differential attacks.

**Table 8:** NPCR and UACI values for the three experiments.

	NPCR (%)			UACI (%)		
	*R*	G	B	*R*	G	B
**Experiment 1**	99.62	99.62	99.61	33.47	99.49	33.45
**Experiment 2**	99.61	99.61	99.61	33.45	33.49	33.48
**Experiment 3**	99.61	99.61	99.60	33.46	33.44	33.48

### MSE and PSNR

For a cipher image to be deemed effective, it must show a substantial deviation from its plain form, ensuring that the encrypted image bears no resemblance to the plain image. The MSE measures the total squared difference between the original and cipher images using the following formula:

22 $$\:{r}_{i,j}=\frac{1}{W\times\:H}{\sum\:}_{i=0}^{W}{\sum\:}_{j=0}^{H}{(P\left(i,j\right)-E\left(i,j\right))}^{2}$$

where $\:P\left(i,j\right)$ represents the pixel value of the plain image while $\:E(i,j)$ denotes the corresponding encrypted pixel value at the position $\:(i,j)$ in the cipher image. The MSE value serves as a criterion for assessing the encryption level of a cryptosystem, where the encryption security level increases with a larger MSE scale. PSNR analysis determines the quality level of encryption; a higher score indicates that the encrypted image closely resembles the original image. Therefore, a smaller PSNR value indicates stronger encryption for a cryptosystem. It can be described as follows:

23 $$\:PSNR=20\times\:{\text{log}}_{10}[255/\sqrt{MSE}]$$

The values of the MSE and PSNR reported in Table 9 highlight the difficulties of recovering the plain image content from the cipher image without knowing the secret decryption key.

**Table 9:** MSE and PSNR values for the three experiments.

	MSE			PSNR		
	*R*	G	B	*R*	G	B
**Experiment 1**	9778	8708	13,492	8.2279	8.7312	6.8298
**Experiment 2**	11,564	9334	15,571	7.4994	8.4298	6.2075
**Experiment 3**	10,625	8716	12,911	7.8672	8.7272	7.0211

### Data loss and data noise

Images experience data loss during transmission or storage. In the case of a cipher cube, some data is lost, and the remaining information is recovered through the decryption process. The experiments partially destroy the cipher cube and then implement the decryption process to get the original images. Images are also affected by noise during transmission or storage, in addition to data loss. To simulate a noise attack, noise was added to the cipher cube at varying intensities of salt and pepper before decrypting the cube. After applying block data loss of 10% and 20%, and under salt-and-pepper noise with densities of 5% and 10%, the proposed algorithm maintained PSNR values ranging from 21.5 dB to 30.2 dB. Corresponding SSIM scores were 0.72 and 0.81, indicating acceptable visual quality and structural preservation despite noise corruption.

**Fig. 11:**
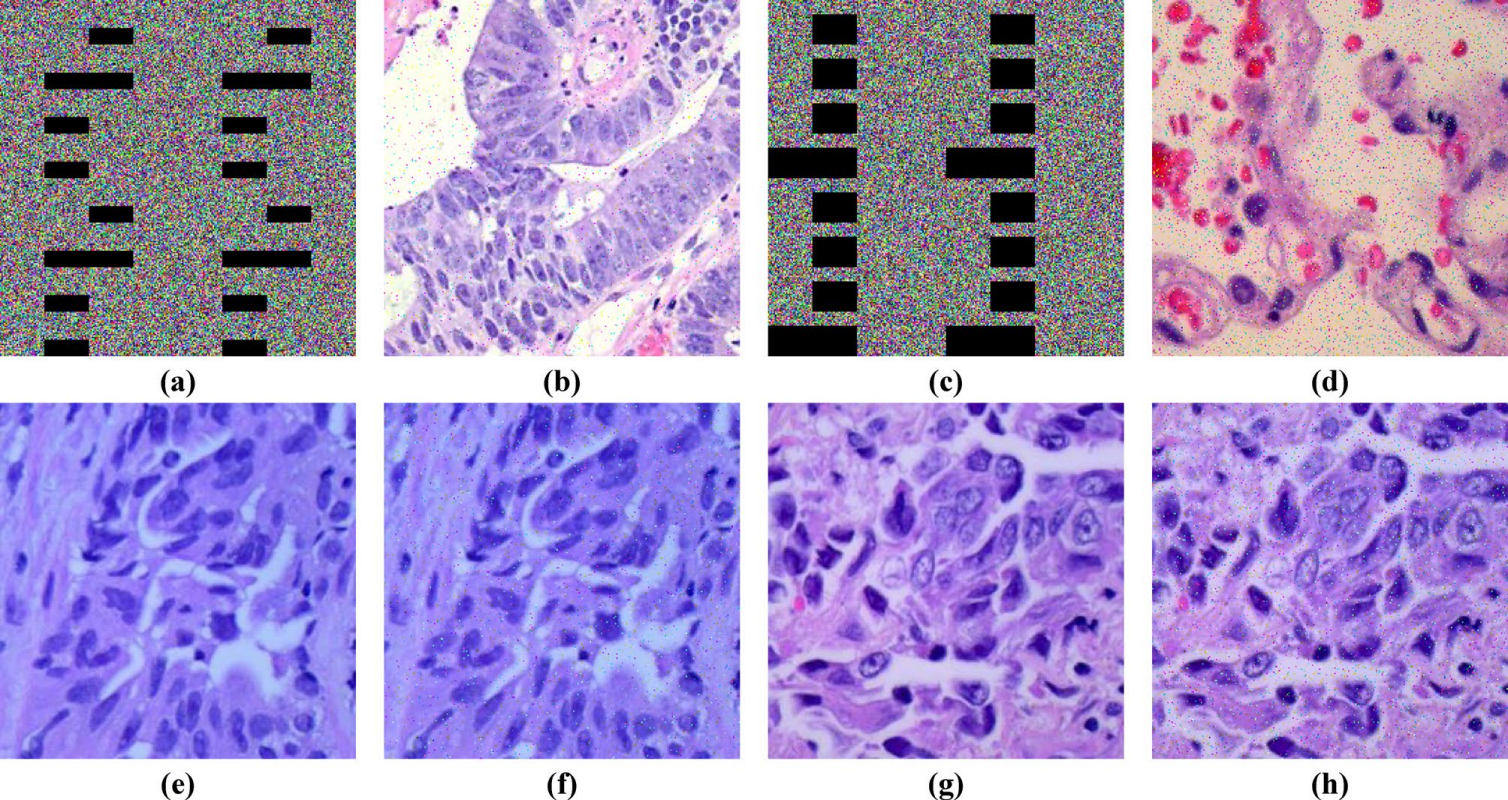
Data Loss **(a**,** c)** Random block loss of more than 10% and 20% of the cipher image, respectively; **(b**,** d)** Corresponding decrypted images. Data Noise **(e**,** g)** Plain images; **(f**,** h)** Decrypted results after adding 5% and 10% salt-and-pepper noise to the encrypted images.

The results are displayed in Fig. 11. The results reveal that the most authentic features of the images have remained intact after decryption, suggesting that the proposed technique can resist these attacks.

### Grayscale images

**Fig. 12:**
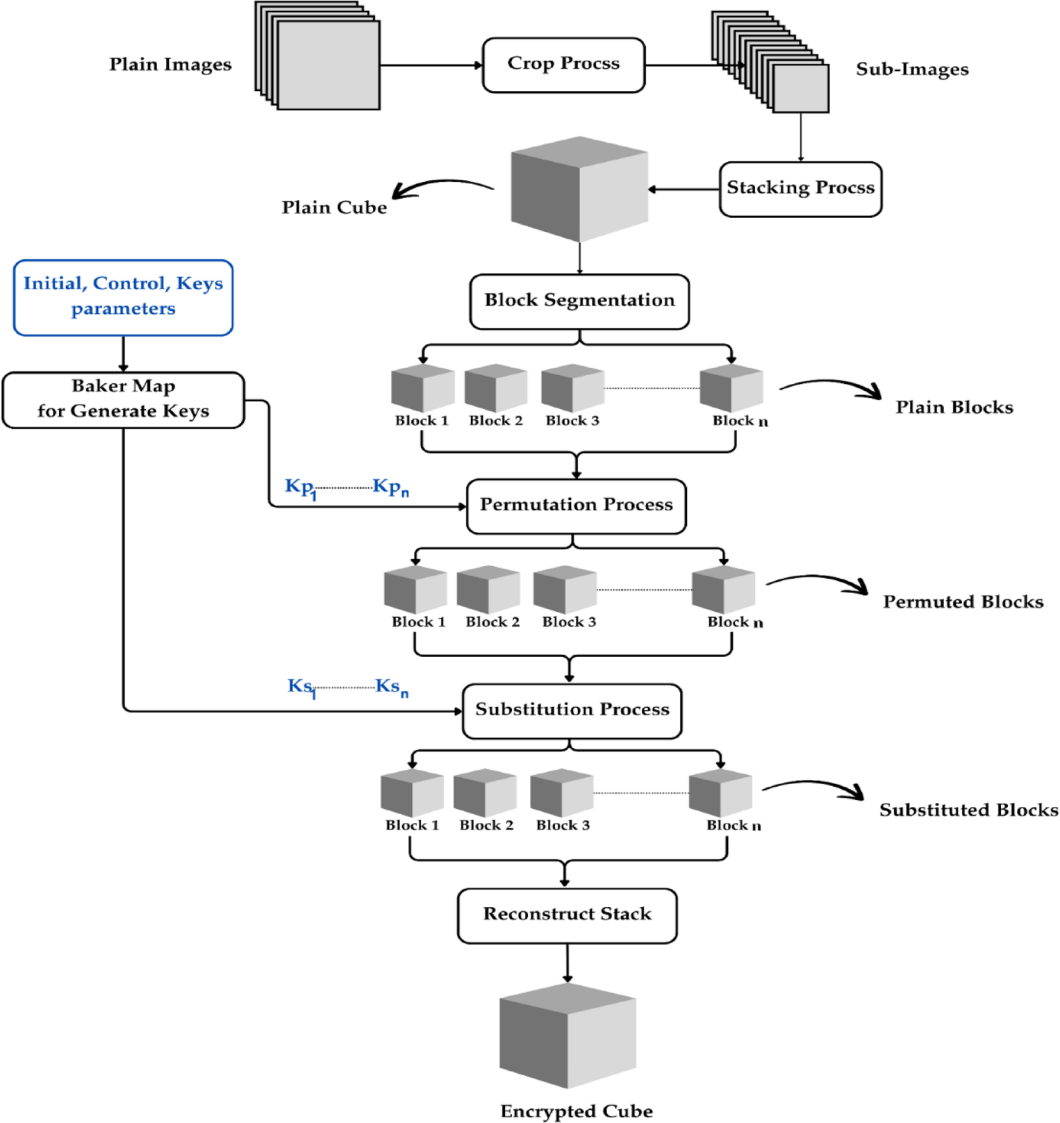
Encryption Process of Grayscale image.

**Fig. 13:**
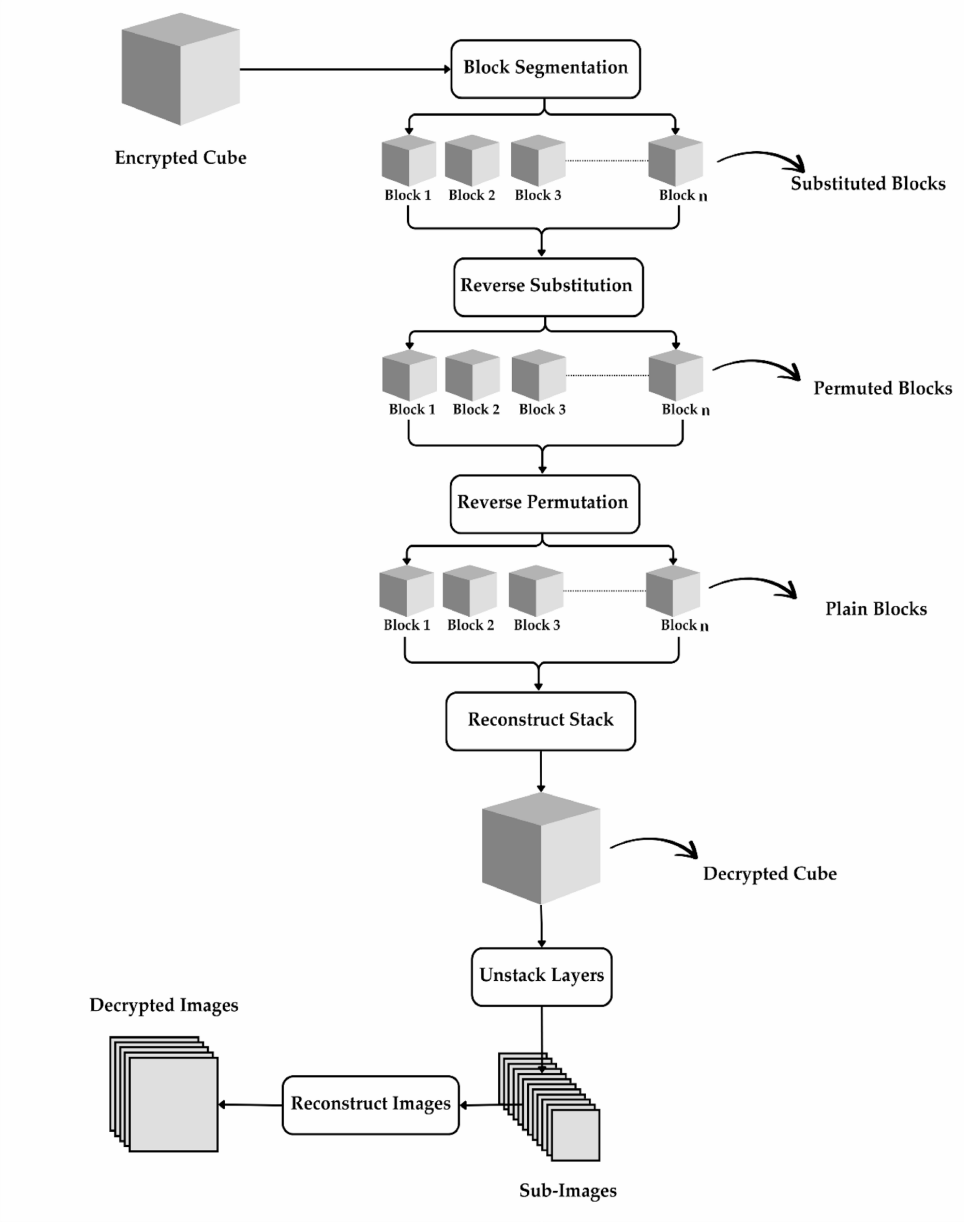
Decryption Process of Grayscale image.

**Fig. 14:**
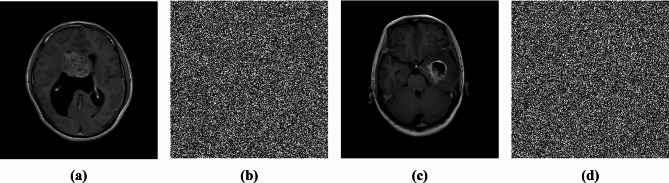
**(a**,** c)** Examples of plain grayscale images and **(b**,** d)** their corresponding encrypted images.

Although this study focuses on color image encryption, the proposed method can also be used for a single grayscale channel. Some analyses are performed to evaluate the scheme for grayscale images. Figures 12 and 13 illustrate the encryption and decryption methodology for grayscale images, and Fig. 14 shows examples of plain grayscale medical images and their corresponding encrypted images.

**Fig. 15:**
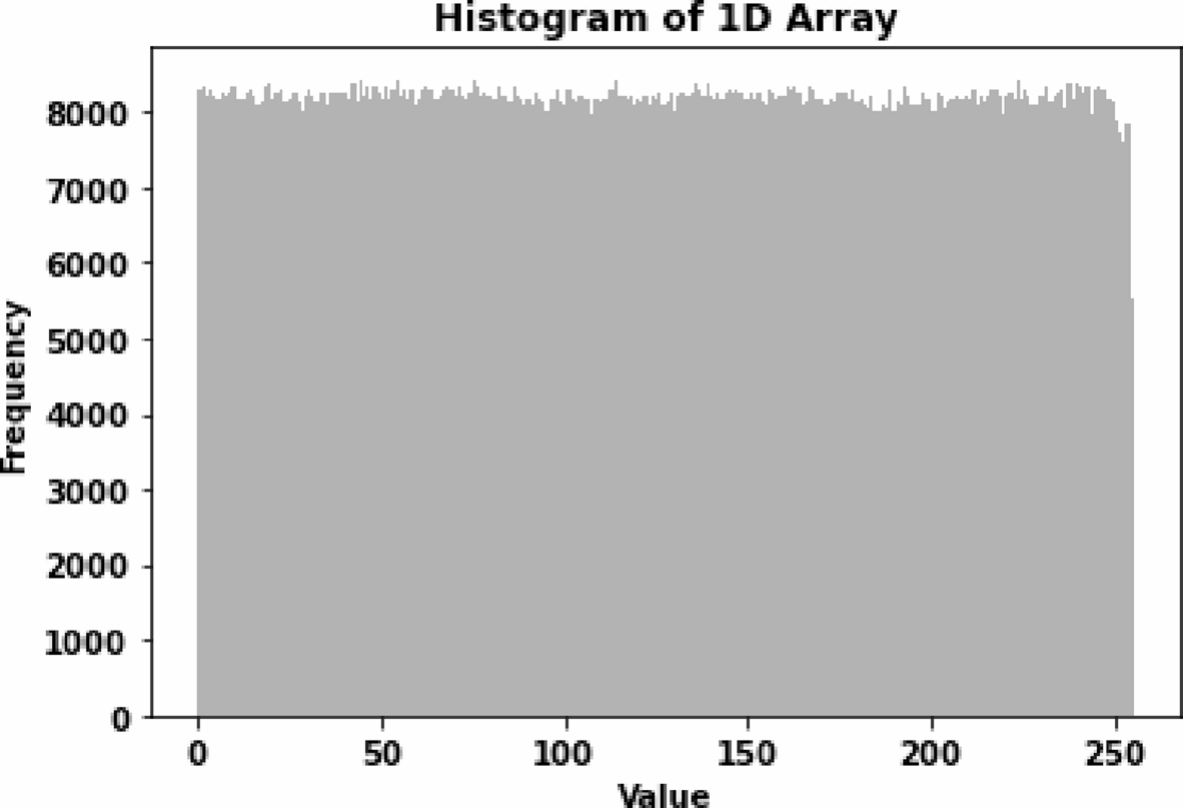
Histogram for 256 × 256 grayscale cipher image.

**Fig. 16:**
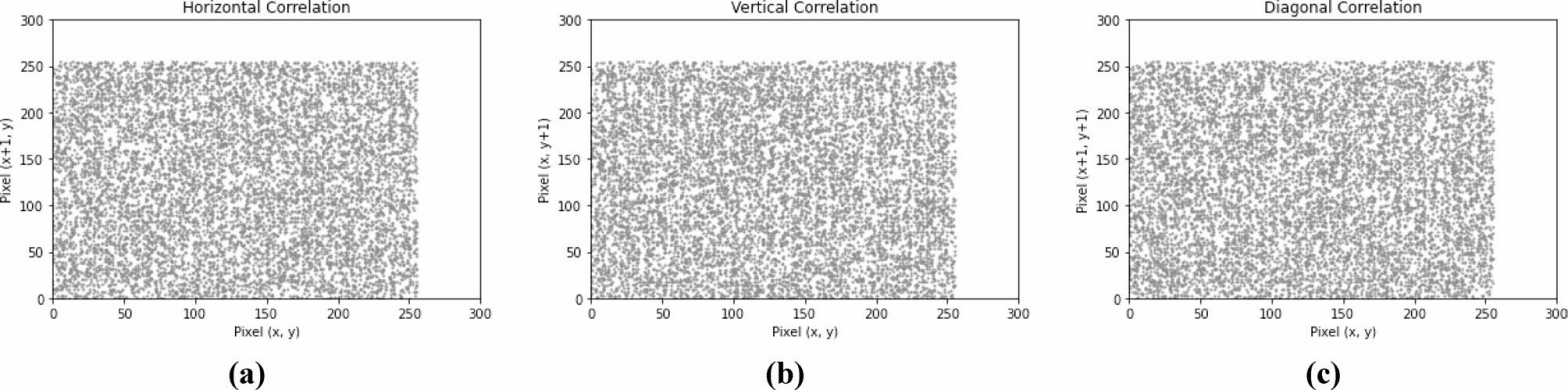
Correlation for 256 × 256 grayscale cipher image in **(a)** Horizontal direction, **(b)** Vertical direction, and **(c)** Diagonal direction.

Figures 15 and 16 show the histogram and correlation analysis results for a 256 × 256 image, demonstrating a uniform distribution. The histogram confirms that pixel intensity values are evenly spread, ensuring the encryption process effectively conceals patterns. Additionally, the reduced correlation between adjacent pixels highlights the proposed method’s strong diffusion and confusion properties, making it resistant to statistical attacks.

Other analyses are shown in Table 10, where the entropy, NPCR, UACI, PSNR, MSE, and execution time demonstrate favorable values. The high entropy value indicates a high level of randomness in cipher images. In contrast, NPCR and UACI values confirm the method’s sensitivity to small changes in plaintext images, ensuring a strong avalanche effect. The PSNR and MSE values also highlight the significant difference between the original and cipher images, reinforcing the encryption strength. Moreover, the execution time further demonstrates the efficiency of the proposed approach, making it suitable for real-time applications.

**Table 10:** Performance analysis.

Images	Entropy	NPCR%	UACI%	MSE	PSNR	Encryption Time (s)
**Image1**	7.99962	99.61	33.48	16,847	5.86548	0.09
**Image2**	7.99958	99.61	33.46	15,785	5.63257	0.1

## Discussion

This study presents a multiple medical image encryption scheme for both color and grayscale images. The proposed approach integrates a chaotic map, 3D transformation, and block segmentation to enhance security and efficiency. Chaotic maps ensure high sensitivity, 3D transformations strengthen confusions and diffusions, and block segmentation optimizes processing speed. Following the trends of hybrid encryption techniques, this scheme effectively balances security and computational performance. Notably, it achieves a 0.1-second encryption time for a 256 × 256-channels image, demonstrating its efficiency for real-time applications. The result highlights the potential of combining chaotic maps with 3D transformation for robust and fast encryptions. The proposed multiple medical image encryption scheme demonstrates superior performance across key security and efficiency metrics.

The algorithm was compared with several recent chaotic-based MIE techniques. Singh et al.^33^ encrypt multiple grayscale images by merging them into an RGB image and applying chaotic permutation and diffusion. The method proposed by Gao et al.^34^ combines three grayscale images into a single image and performs encryption on a single channel using a chaotic system. The algorithm by Zhang and Zhang^35^ encrypts multiple images using chaotic scrambling and gene fusion based on DNA operations. These algorithms were selected for comparison as they represent MIE approaches built entirely on chaotic principles.

Additionally, the scheme was evaluated against MIE algorithms that employ alternative strategies. Xu et al.^36^ introduced an approach that encrypts multiple medical images by scrambling the region of interest (ROI) and applying diffusion using odd-even interleaved points. It represents a domain-specific MIE method that focuses on protecting sensitive content. Xu^37^ proposed a multiple-image encryption algorithm based on orthogonal arrays with strength 3, incorporating chaotic operations to perform spatial permutation and substitution across images. Ye and Guo^38^ presented an MIE algorithm that embeds encrypted images into a carrier image using 3D-DCT and a 3D chaotic map, producing a visually meaningful encrypted result with combined encryption and hiding. These works were selected for comparison as they present recent MIE techniques with varied strategies.

Further comparison was made with hybrid techniques that integrate chaotic systems with classical cryptographic or transformation elements. Zhang and Liu^23^ introduced a stereo Zigzag transformation to expand 2D scrambling into 3D, combined with hash-based chaotic diffusion. Alexan et al.^39^ developed an MIE algorithm using SVD, modified RC5, and a hyperchaotic Hill cipher for satellite imagery. Xu et al.^40^ proposed an approach based on a novel chaotic system and odd-even interleaving strategy for encrypting medical images. These methods were chosen for comparison because they represent recent hybrid MIE approaches that target enhanced robustness and domain-specific performance.

As shown in Table 11, the proposed algorithm achieves faster execution times than most recent MIE methods. The encryption time of 0.1 s for a 256 × 256-channel image makes it more efficient than both chaotic-only and transform-based approaches.

Security analysis reveals that the scheme maintains high entropy, ensuring a uniform distribution of ciphertexts. It provides a large key space, making brute-force attacks infeasible. The encryption process effectively reduces pixel correlation, enhancing security against statistical attacks. Table 12 presents the different MIE experiment key space and entropy results obtained using our scheme and recent algorithms, as well as various experimental correlation results.

**Table 11:** Encryption time comparison.

Algorithm	Device Properties	Average time for each image (s)
**Proposed**	Personal Computer, Intel Core i5-9300 H @ 2.40 GHz, 8GB RAM, Windows 11.	0.11
**Ref. [33]**	Wolfram Mathematica 13, Fujitsu Celsius Workstation, Intel Xeon W-2133 @ 3.60 GHz, 32GB RAM	0.25
**Ref. [34]**		0.094
**Ref. [35]**	MATLAB R2018a, Personal Computer, Intel Core i5 @ 1.80 GHz, 8GB RAM, Windows 10 (64-bit)	0.31
**Ref. [36]**	Personal Computer, Intel Core i7-10750 H @ 2.60 GHz, 16GB RAM	0.14
**Ref. [37]**		0.20
**Ref. [38]**	MATLAB 2020a, Personal Computer, AMD R7 5800 H @ 3.20 GHz, 16GB RAM	0.15
**Ref. [23]**	Personal Computer, 2.8 GHz CPU, 8GB RAM, Windows 10 (64-bit)	0.25
**Ref [39]**	Personal Computer, Intel^®^ Core™ i7-7500U CPU operating at 2.7 GHz and equipped with 8 GB of RAM	0.23

**Table 12:** Key space, entropy, and correlation comparison.

	Key space	Entropy	Correlations		
			H	V	D
**Proposed**	$\:{2}^{1994}$	7.9999	−0.00001	−0.00050	0.00039
**Ref. [33]**	$\:{2}^{332}$	7.9994	0.00179	−0.01642	−0.00551
**Ref. [34]**	$\:{2}^{624}$	7.9994	0.0021	0.0029	0.0023
**Ref. [35]**	$\:{2}^{390}$	7.9995	−0.0029	−0.0049	−0.0021
**Ref. [36]**	$\:{2}^{512}$	7.9994	0.0015	0.0011	0.0015
**Ref. [37]**	$\:{2}^{186}$	7.9993	− 0.01469	0.00231	0.00153
**Ref. [38]**	$\:{2}^{149}$	ــــ	−0.0019	− 0.0018	− 0.0121
**Ref. [23]**	$\:{2}^{442}$	7.9997	−0.0007	0.0002	0.0010
**Ref. [39]**	$\:{2}^{10524}$	7.9991	−0.00392	−0.00247	−0.0066
**Ref. [40]**	$\:{2}^{512}$	7.9994	0.0058	0.0062	0.0024

Additionally, the scheme achieves high NPCR (~ 99%) and UACI (~ 33%), indicating strong sensitivity to small changes in plain data. Image quality metrics further validate the scheme’s effectiveness. A high MSE and low PSNR confirm strong encryption, ensuring encrypted images appear noise-like and unreadable. These improvements make the proposed approach well-suited for secure and efficient multiple medical image encryption. The values of UACI, NPCR, MSE, and PSNR for different algorithms are listed in Table 13.

**Table 13:** NPCR, UACI, MSE, PSNR analysis comparison.

	NPCR (%)	UACI (%)	MSE	PSNR
**Proposed**	99.61	33.47	12,156	7.3789
**Ref.[33]**	99.62	33.42	ــــ	8.50339
**Ref.[34]**	99.61	33.46	ــــ	ــــ
**Ref.[35]**	99.64	33.38	ــــ	ــــ
**Ref.[36]**	99.61	33.46	ــــ	ــــ
**Ref.[37]**	99.61	33.47	8261	8.9605
**Ref.[38]**	99.61	33.46	ــــ	ــــ
**Ref.[23]**	99.59	33.42	ــــ	ــــ
**Ref.[39]**	99.61	29.49	8336.25	8.9211
**Ref. [40]**	99.61	33.47	ــــ	ــــ

The proposed encryption algorithm is based on chaotic systems and block-wise permutation–diffusion operations rather than number-theoretic or algebraic primitives. As a result, it is not directly susceptible to well-known quantum algorithms such as Shor’s algorithm, which targets RSA and ECC, or Grover’s algorithm, which speeds up key search in symmetric cryptosystems. Since the chaotic sequences used for scrambling and diffusion are dynamically generated per block and do not rely on fixed key scheduling or algebraic structure, the algorithm provides an inherent level of resistance to existing quantum attack models.

However, a formal quantum cryptanalysis of chaos-based image encryption schemes remains an open area of research. At present, there are no known quantum algorithms that can efficiently break such systems. Nevertheless, evaluating their security under quantum computing assumptions is essential in the context of post-quantum cryptography and will be addressed in future work.

## Conclusion

A multiple-image encryption algorithm for medical images is proposed based on stack representation and block segmentation. In the preprocessing phase, multiple medical grayscale images are cropped into sub-images and stacked into a 3D cube to prepare for encryption. The cube then undergoes block segmentation, where each block is individually subjected to confusion and diffusion processes. These processes leverage keys generated from a Baker chaotic map, employing swapping and XOR operations to produce a fully encrypted cube. The proposed algorithm’s effectiveness is evaluated through comprehensive experiments, demonstrating its potential as a secure solution for encrypting medical grayscale images. By integrating image transformation, sub-image stacking, block segmentation, and the confusion and diffusion phases, the algorithm achieves high encryption strength and robustness against attacks. Performance analyses further reveal excellent encryption speed and strong security characteristics, making it a promising approach for medical image encryption.

## Data Availability

The data supporting this study’s findings are available from the corresponding author upon request.

## Abbreviations

MIE: Multiple Image Encryption
DNA: Deoxyribonucleic acid
AI: Artificial Intelligence
DCT: Discrete Cosine Transform
NJTC: Nonlinear Joint Transform Correlator
GT: Gyrator Transform
JGPD: Joint Gyrator Power Distribution
SZT: Stereo Zigzag Transformation
ZT: Zigzag Transformation
ESC map: Exponent Sine-Cosine map
RGB: Red, Green, Blue
SIR: Stack Image Representation
2D: Two Dimensional
3D: Three Dimensional
OS: Operating System
UACI: Unified Average Changing Intensity
NPCR: Number of Pixels Change Rate
MSE: Mean Square Error
PSNR: Peak-to-nose ratio
